# A Loose Screw: An Open Surgical Approach for Removing Large Laryngotracheal Foreign Bodies

**DOI:** 10.7759/cureus.116019

**Published:** 2026-09-09

**Authors:** Hussein M Saleh, Anya Costeloe, Anthony Howard, Prasad John Thottam

**Affiliations:** 1 College of Osteopathic Medicine, Michigan State University, East Lansing, USA; 2 Facial Plastic and Reconstructive Surgery, Bella Cosmetic Surgery, Washington, DC, USA; 3 Otolaryngology - Head and Neck Surgery, Corewell Health William Beaumont University Hospital, Royal Oak, USA; 4 Allergy and Immunology, Children's Hospital of Michigan, Detroit Medical Center, Detroit, USA

**Keywords:** airway foreign body, cricoid split, foreign body aspiration, pediatric otolaryngology, rigid bronchoscopy, tracheostomy

## Abstract

Unusual airway foreign bodies have long interested airway specialists, and in the present case, the unusual feature is not the type of foreign body but its size. We describe the surgical management of a large, high-risk airway foreign body in a pediatric patient when an endoscopic approach is unsuccessful. A 22-month-old male aspirated a 3.5-inch drywall screw. The size of the screw in the airway of a patient of this age prompted a multidisciplinary approach to surgical removal, and we detail the preoperative, operative, and postoperative care of this patient, focusing on the surgical technique used and the coordination required among Pediatric Otolaryngology, Pediatric Surgery, and Pediatric Anesthesiology. Modern bronchoscopic techniques have made the removal of airway foreign bodies routine, but large airway foreign bodies remain a difficult challenge, and at times, an open technique is required. Traditional open surgical approaches, including thoracotomy, bronchotomy, and pulmonary resection, carry high rates of morbidity. Extended tracheostomy with cricoid split is a potential option for the removal of large laryngotracheal foreign bodies and merits consideration before proceeding to thoracotomy.

## Introduction

Foreign body aspiration remains a leading cause of death from unintentional injury in children under one year of age and continues to represent a meaningful source of morbidity throughout childhood [1]. Toddlers are disproportionately affected because of incomplete dentition, immature swallow coordination, a small-caliber airway, and a developmental tendency to explore objects orally [1]. In a meta-analysis of 174 published case series, Foltran F et al. found that nearly 20% of children with an inhaled foreign body were between birth and three years of age and that acute or chronic complications occurred in approximately 15% of patients [2]. Organic material accounts for the majority of reported aspirants, but non-food objects remain a substantial minority and are disproportionately represented among cases in which standard endoscopic retrieval fails [1].

Rigid bronchoscopy is the established first-line intervention and succeeds in most cases. In a review of 12,979 pediatric bronchoscopies, Fidkowski CW et al. reported that 88% of foreign bodies lodged in the bronchial tree, with the remainder arrested in the larynx or trachea. That review documented a procedural mortality rate of 0.42% and a composite major complication rate of 0.96%, encompassing severe laryngeal edema or bronchospasm requiring tracheotomy or reintubation, pneumothorax, pneumomediastinum, cardiac arrest, tracheal or bronchial laceration, and hypoxic brain injury [3]. Only a small minority of patients cannot be managed endoscopically. In a review of 6,693 reported cases, Marks SC et al. identified thoracotomy in 2.4% and tracheostomy in 1.6%, with tracheostomy most often performed for postoperative airway protection rather than for retrieval itself [4].

We would propose, though no published criterion defines this, that when the greatest dimension of an object approaches or exceeds the distance from the carina to the glottis, that object cannot be reoriented into an axis that will clear the vocal folds. A conventional tracheotomy sited at the second or third tracheal ring may provide an aperture that is both too caudal and too small to deliver it. In this circumstance, the surgeon is left to choose between thoracotomy with bronchotomy or pulmonary resection and an improvised open cervical approach.

We report a 22-month-old male who aspirated a 3.5-inch drywall screw that came to rest with its head on the carina and its threaded tip in the subglottis. After failed endoscopic retrieval and an unsuccessful attempt through a standard tracheostomy, the stoma was extended superiorly through the first and second tracheal rings and then through the cricoid cartilage, after which the screw was rotated free and delivered. We describe the operative rationale, the airway sequelae, and where we believe this maneuver may fit within the management algorithm.

## Case presentation

A 22-month-old male was placed in his crib for a time-out after fighting with his brother at home. He was unsupervised in his crib for 10 to 20 minutes. His stepmother went to the room when he began crying violently and found him in distress, crying, with a small amount of blood-tinged saliva at his mouth. She notified her husband and called EMS, and the child was brought to the hospital, where a radiograph was obtained. This radiograph demonstrated a large screw that was thought to lie within the esophagus based on its size and position. At the time, this correlated with the clinical picture, as the patient was stable, breathing easily without stridor, and playful with the ED staff. Otolaryngology evaluated the patient in the ER at the community hospital and recommended transfer to a tertiary care center, where the child could receive appropriate subspecialty support. The patient was transferred to our hospital for surgical evaluation and a higher level of care.

Shortly after arrival at our hospital, the child developed nasal flaring, intercostal retractions, and biphasic stridor. Chest radiography was repeated and demonstrated that the large screw was, in fact, within the trachea (Figure 1). Pediatric Surgery was consulted, and the patient was consented for rigid bronchoscopy and removal of the foreign body. The pediatric surgery team performed the initial direct laryngoscopy and attempted removal of the foreign body but was unable to pass it through the subglottis and glottic introitus. The team converted to an open approach at that point based on several converging considerations. The screw was longer than the distance from the carina to the glottis, so no degree of axial rotation would allow the tip to clear the glottic inlet, making the failure one of fixed geometry rather than instrumentation or technique. Cephalad traction on the head of the screw drove the threaded tip against the posterior pharyngeal wall, so continued force risked mucosal laceration or pharyngeal perforation. The threads were engaged with the airway mucosa along their length, and the object was of a caliber that would completely occlude the trachea if control were lost during withdrawal. Progressive laryngeal and tracheal edema was expected with each successive attempt, narrowing the margin of safety for both further endoscopy and any subsequent airway intervention. On this basis, the pediatric surgeon converted to an open surgical approach with a tracheostomy.

**Figure 1:**
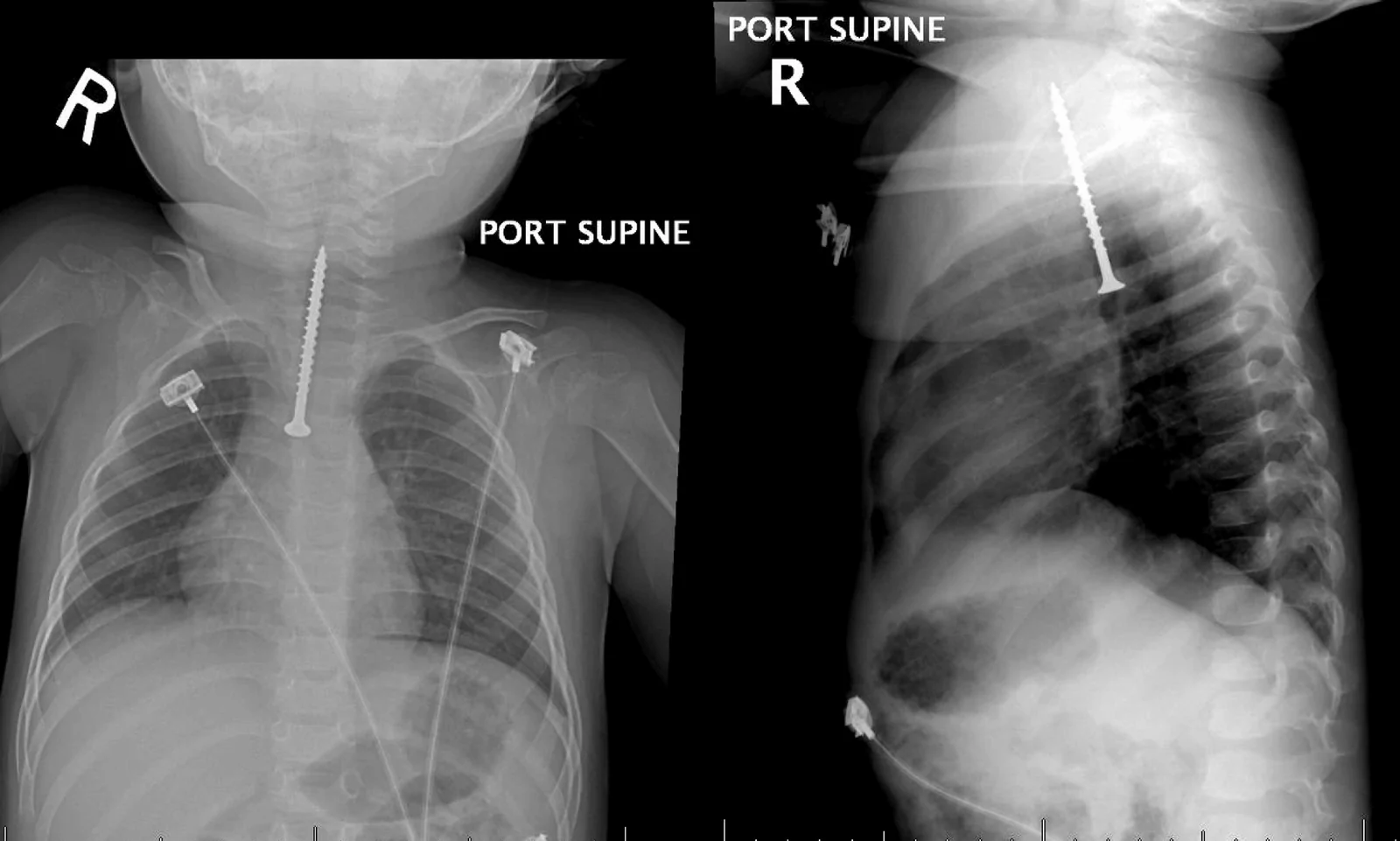
Anteroposterior (left) and lateral (right) chest radiographs demonstrating the foreign body within the trachea, with the head of the screw resting inferiorly on the carina and the tip of the screw positioned superiorly in the subglottis.

Pediatric Otolaryngology was called into the room, where the foreign body was noted to be lodged above the tracheostomy and below the vocal folds. The length of the foreign body proved to be the restrictive factor. With the head of the screw grasped and cephalad traction applied, the glottis acted as a fulcrum, and the superior tip was driven posteriorly against the pharyngeal wall near the nasopharynx rather than advancing toward the oral cavity. Delivery through the mouth would have required a rigid object of this length to clear the glottis and then negotiate the oropharyngeal angle, which was not possible in a patient of this age, and the threaded tip could not be used as a point of traction without lacerating the pharyngeal mucosa. The stoma was extended superiorly rather than proceeding to thoracotomy because the obstruction to delivery was the cricoid ring and upper tracheal rings, and a longitudinal aperture in the anterior airway addressed that constraint directly with far less morbidity. Using a mini Beaver blade, a midline incision was made from the superior aspect of the tracheostomy stoma and carried through the second and first tracheal rings. The foreign body still could not be delivered through the extended stoma, and the incision was extended further superiorly through the cricoid cartilage. At this point, the head of the screw could be angled out of the extended incision without the tip damaging the posterior pharyngeal wall. Even with the cricoid split, the foreign body required gentle counterclockwise rotation to unscrew it from the airway (Figure 2). After successful removal, a laryngoplasty was performed, and the cricoid cartilage was reapproximated with polydioxanone (PDS) suture, ensuring adequate alignment of the perichondrium. The airway was then assessed and demonstrated severe laryngeal and tracheal edema. The tracheostomy was revised, and a pediatric tracheostomy cannula was left in place (Figure 3).

**Figure 2:**
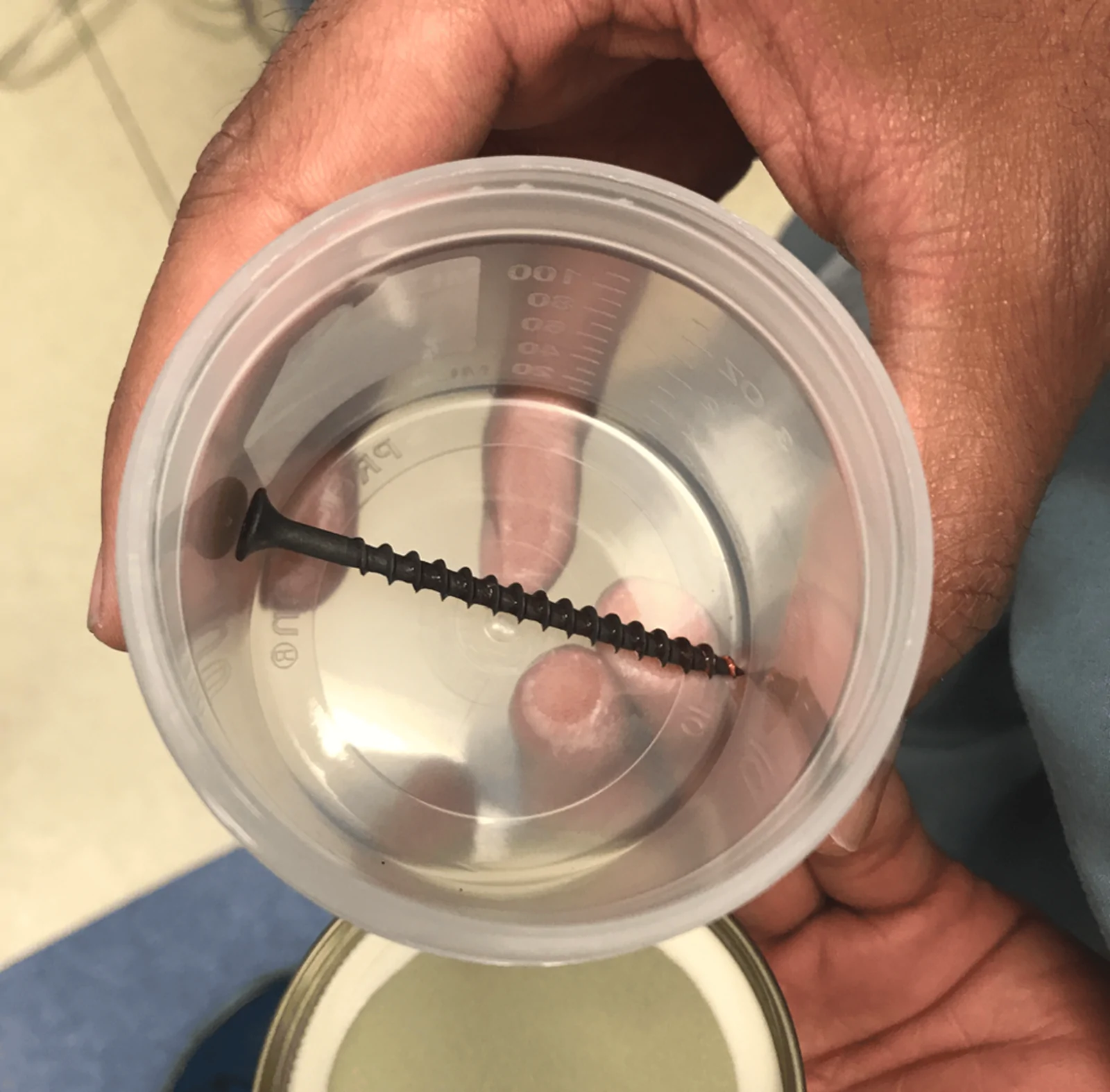
The 3.5-inch drywall screw after removal via the open technique.

**Figure 3:**
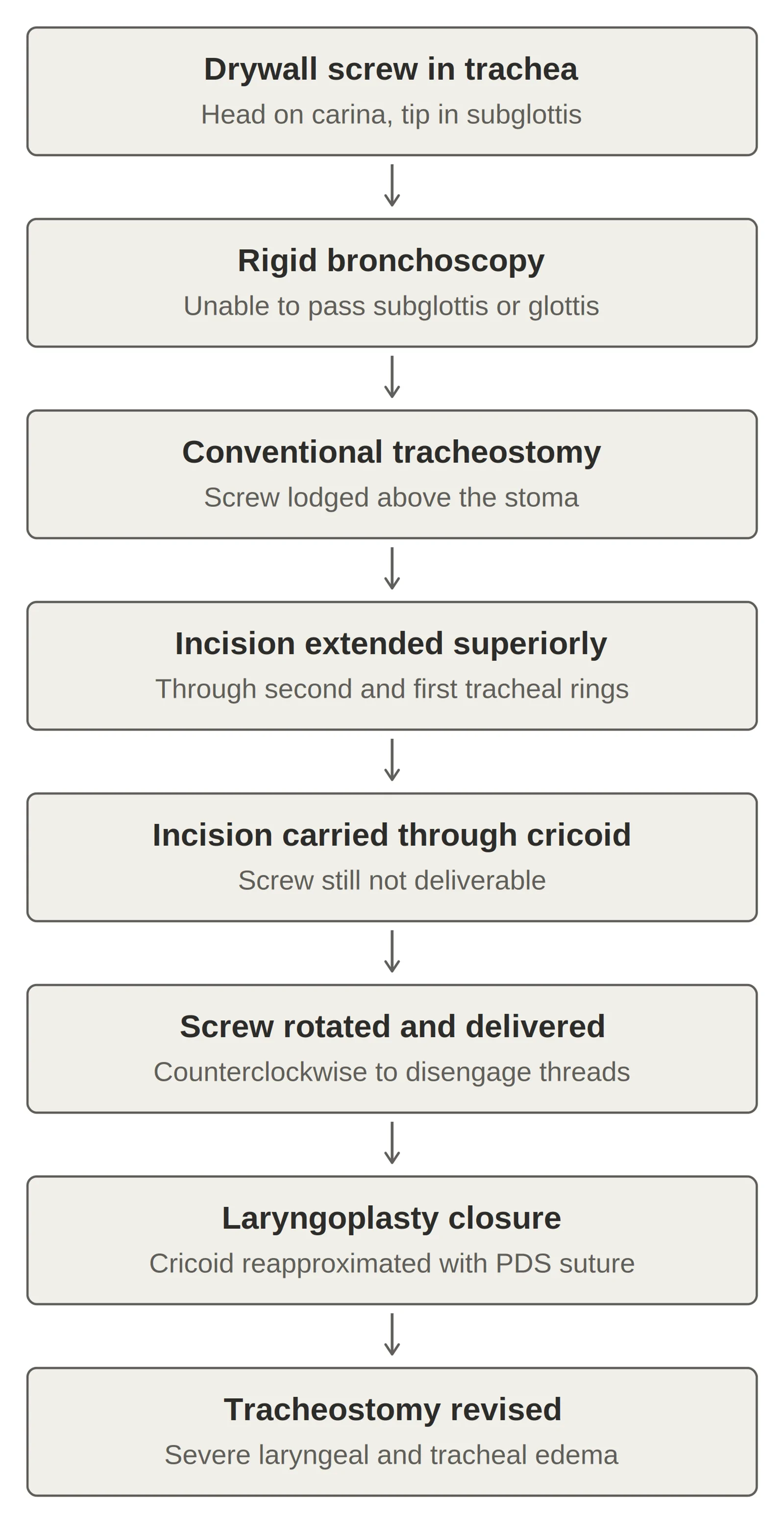
Operative sequence.

Postoperatively, the patient recovered quickly without functional deficit. Prior to discharge, he underwent direct laryngobronchoscopy with tracheostomy tube change. At that time, he was noted to have Myer-Cotton grade III subglottic stenosis, and the subglottis was injected with steroid. At his next direct laryngobronchoscopy four weeks later, a small amount of granulation tissue above the tracheostomy stoma was removed, and the airway was resized. His subglottic stenosis had improved significantly and was Myer-Cotton grade I. Three months after the initial event, he was successfully decannulated and was doing well. Written informed consent for publication of this case and the accompanying images was obtained from the patient’s parent or legal guardian.

## Discussion

Screws are not uncommon aspirants. Foltran F et al. grouped screws with pins, nails, and tacks, a category that accounted for 7.6% of all reported airway foreign bodies [2]. What distinguishes the present case is not composition but dimension. A 3.5-inch object within the airway of a 22-month-old spanned essentially the entire length of the trachea, resting on the carina inferiorly and reaching the subglottis superiorly.

The initial radiographic interpretation deserves comment. At the referring facility, the screw was read as esophageal, in part because an object of that size seemed anatomically incompatible with a tracheal position, and the child reinforced that impression by remaining comfortable, playful, and free of stridor. Symptoms did not appear until after transfer. Two points follow. First, in a small child, a radiodense linear object of this length should be presumed to occupy the airway until definitively proven otherwise, since the trachea is the more dangerous of the two possibilities and the more difficult to exclude on a single view. Second, the absence of stridor at presentation does not exclude tracheal impaction, particularly when the object is oriented along the axis of the airway and is not yet producing a critical reduction in cross-sectional area.

As described above, the failure of endoscopic retrieval in this case was a problem of geometry rather than technique or instrumentation, and this distinction is what justified early conversion. Where endoscopic failure reflects instrument selection, grasping angle, or object orientation, additional attempts are reasonable. Where the greatest dimension of the object exceeds the distance from the carina to the glottis, no number of further attempts will succeed, and each one adds mucosal trauma and edema without prospect of benefit. Recognizing that distinction early is, in our view, the single most useful judgment in these cases.

Open removal of airway foreign bodies is well described but uncommon. Marks S et al., reviewing 300 cases over 30 years at their own institution, required an open technique in only two and, in their broader literature review, found 161 thoracotomies and 104 tracheostomies among 6,693 patients. Of those 104 tracheostomies, 12 were created as a conduit for the bronchoscope, 11 for ventilatory assistance, and 10 specifically to assist in the delivery of large foreign bodies [4]. Singh JK et al. reported tracheostomy in only four of 342 children managed for airway foreign body obstruction and similarly framed the indication in terms of object size and airway protection [5]. Fraga JC et al. removed a foreign body too large to pass the subglottis in an 8-year-old through a tracheotomy under bronchoscopic control, closing the tracheal opening primarily without leaving a cannula [6]. Yang X et al. described a 16-month-old girl with a 9-cm sharp metallic chain in the lower trachea and right mainstem bronchus, which was delivered through a low tracheotomy while ventilation was maintained through a rigid bronchoscope positioned proximally [7]. Wang HC et al. assembled a case series and literature review of tracheostomy-assisted retrieval and reached comparable conclusions regarding indications [8]. Ulkü R et al. made the analogous argument for aspirated pen caps, a foreign body whose wide caliber similarly defeats endoscopic delivery through the glottis [9].

These reports share a common structure. In each, the object is delivered through a tracheal window created at or below the level of impaction, and in each, the technique succeeds because the foreign body lies within the trachea proper. Our case differed in a way that rendered that approach insufficient. The screw straddled the subglottis, and its superior end lay above the level of any tracheal opening that could reasonably be created. A window in the trachea, however generous, could not deliver an object whose upper pole was fixed against the posterior pharyngeal wall above it. The obstruction was not the tracheal wall but the cricoid ring.

This observation is the rationale for the technique we describe. The cricoid is the only complete cartilaginous ring in the airway and constitutes the narrowest point of the pediatric larynx; it therefore functions as the fixed constraint on any object being withdrawn from the trachea in a cephalad direction. Extending the tracheostomy superiorly through the second and first tracheal rings converted a circular stoma into a longitudinal aperture, and carrying that incision through the cricoid released the constraint entirely. The anatomic maneuver is not itself new. The anterior cricoid split was described by Cotton RT and Seid AB in 1980 as an alternative to tracheotomy in premature infants who failed extubation [10] and was subsequently extended by Seid and Canty to older infants and children with acquired subglottic injury [11]. We employed the cricoid split not to enlarge a stenotic subglottis but to create an exit through which an impacted foreign body could be delivered. Even with the cricoid divided, the screw required gentle counterclockwise rotation to disengage its threads from the airway mucosa, a detail we would emphasize to any surgeon encountering a threaded foreign body. The cartilage was then reapproximated with PDS suture in a laryngoplasty closure with careful attention to perichondrial alignment, and given the severity of the observed laryngeal and tracheal edema, a pediatric tracheostomy cannula was left in place.

The airway sequelae of this approach should be stated plainly. At direct laryngobronchoscopy prior to discharge, the patient demonstrated grade III subglottic stenosis by the Myer-Cotton classification [12], which was managed with intralesional steroid injection. At repeat endoscopy four weeks later, a small amount of suprastomal granulation tissue was removed, the airway was resized, and the stenosis had improved to grade I. The child was decannulated three months after the initial event and has done well without functional deficit.

Four factors plausibly contributed to the observed narrowing, and their relative contributions cannot be separated in a single case. The foreign body itself was a threaded metallic object impacted across the subglottis. Its threads were engaged with the mucosa along their length, producing circumferential abrasion at precisely the level where the stenosis was subsequently observed. Repeated instrumentation during attempted endoscopic retrieval delivered additional mucosal trauma to the same segment. Laryngeal and tracheal edema was severe at the conclusion of the case and was itself the consequence of the preceding two factors. The anterior cricoid split divided the only complete cartilaginous ring in the airway, and division of the cricoid carries an inherent risk of subsequent narrowing, although the cartilage was reapproximated with attention to perichondrial alignment rather than left open as in the original description of the technique [10].

The time course argues that the reversible components predominated. Mature cicatricial stenosis does not remit over four weeks, whereas edema and granulation tissue do, and both responded to intralesional steroid and endoscopic debridement. Had structural narrowing from the cricoid division been the principal mechanism, we would expect a fixed caliber reduction that persisted or progressed rather than improving from grade III to grade I within a month and permitting decannulation at three months. We would therefore caution against attributing the stenosis primarily to the cricoid split. What can be said is that the observed morbidity was transient and fully reversible and that it compares favorably with the alternative. In the Marks series, 102 of 161 thoracotomies involved bronchotomy and 59 involved pulmonary resection, and two deaths were attributed to thoracotomy performed for airway foreign body [4].

The conduct of this case also argues for early assembly of a multidisciplinary team. Pediatric Otolaryngology, Pediatric Surgery, and Pediatric Anesthesiology each contributed to the eventual outcome, and the decision to convert to an open approach was made intraoperatively under conditions in which delay would have been consequential. In a shared airway of this kind, ventilation must be maintained around or through the foreign body while the surgical field is actively being manipulated, and the anesthetic plan must anticipate both the possibility of complete obstruction and the possibility of conversion to an open neck. Where a large laryngotracheal foreign body is identified preoperatively, we would recommend that a tracheostomy set be opened, that thoracic surgical support be available, and that the operative team explicitly discuss the threshold for abandoning endoscopic retrieval before the case begins.

This report carries the limitations inherent to a single case. We cannot estimate how frequently this technique would be required, nor can we compare its morbidity against alternative approaches in any statistical sense, and follow-up is limited to the interval through decannulation without long-term data on swallowing, voice, airway growth, or restenosis. With those constraints acknowledged, we would suggest where this maneuver may fit. When endoscopic retrieval of a laryngotracheal foreign body fails because the object is too long to clear the glottis, and when a conventional tracheostomy provides insufficient access, extension of the stoma superiorly through the upper tracheal rings and cricoid with subsequent laryngoplasty closure represents a potential option that can be considered before proceeding to thoracotomy.

## Conclusions

Modern bronchoscopic techniques have made the removal of airway foreign bodies a routine procedure. Large airway foreign bodies, however, remain a difficult challenge, and sometimes an open technique is required. When preparing for the removal of a large foreign body in a pediatric patient, a multidisciplinary team should be assembled, ideally consisting of Pediatric Otolaryngology, Pediatric Surgery, and Pediatric Anesthesiology, and the surgeon should be prepared to convert to an open technique should the endoscopic attempt fail. Traditional open surgical approaches, including thoracotomy, bronchotomy, and pulmonary resection, have been associated with increased morbidity. Extended tracheostomy with cricoid split is a potential option for the removal of large laryngotracheal foreign bodies and merits consideration before proceeding to thoracotomy. Additional reported experience will be required to determine its broader applicability.
